# Surface Roughness of Composite Panels as a Quality Control Tool

**DOI:** 10.3390/ma11030407

**Published:** 2018-03-09

**Authors:** Onur Ulker

**Affiliations:** 1 Department of Interior Architecture and Environmental Design, University of Kırıkkale, 71450 Yahşihan, Turkey; oulker@okstate.edu or ulker79o@hotmail.com; Tel.: +1-405-385-3104; 2 Department of Natural Resource Ecology and Management, Oklahoma State University, Stillwater, OK 74078-6013, USA

**Keywords:** surface roughness, composites, hardness, overlaying

## Abstract

This paper describes a study of the quantify surface roughness of experimentally manufactured particleboards and sandwiched panels having fibers on the surface layers. Surface quality of specimens before and after being overlaid with thin melamine impregnated papers was determined by employing profilometer equipment. Roughness measurements and Janka hardness were carried out on the specimens conditioned at 60% and 95% relative humidity levels. Based on the findings in this work, surface roughness of the specimens that were exposed two relative humidity exposure showed significant differences from each others. Data determined in this study could be beneficial to understand behavior of such panels exposed different humidity levels.

## 1. Introduction

In manufacture value-added products, wood based panels, such as fiberboards and particleboards, are used extensively as underlayment for thin overlays. These overlaid panels are main members in production of furniture and kitchen cabinets. As a well-known fact that wood-based composites are hygroscopic material and their dimensional stability varies with fluctuating as function of relative humidity. Especially, their surface quality depending on particle or fiber size on the face layer plays an important role on. Overall surface quality of panel is important so-called telegraphing effect of the roughness of the substrate shows through the overlay. Therefore, it is necessary to determine not only surface of the panel but also roughness of overlaid member with changing relative humidity so that they can be used more efficiency for different types of applications. 

Laminated and overlaid particleboard (PB) and medium density fiberboard (MDF) have been commonly used manufacturing indoor cabinets in Europa and the United States of America (USA) for over 35 years [1,2]. Overlaid wood composites consisted of two main layers. These layers are resistant decorative paper and composite panels. Currently there is no an official standard used to analyze the surface properties of wood composite panels (PB, MDF) [2,3,4,5,6,7]. The most commonly used method to evaluate the surface quality of composites is the stylus method, which provides well accepted numerical values [8,9,10,11]. 

If surface irregularities on a composite panel are present, they may show through the overlays. Therefore, the overall quality of the final product can be affected by such irregularities on the substrate. In several previous studies, the stylus technique has been used to calculate the value of surface roughness of wood composites [12,13,14,15]. Surface roughness and surface stability of different type of panels were also investigated another work [16,17]. In one of the recent studies, it was found that surface roughness of laminated high-density fiber board (HDF) did not affect the surface quality of samples [1,2]. 

In another previous work, medium density fiberboard surface roughness values were calculated using a fine stylus technique. During the sanding of the panels, surface roughness variations are introduced. In order to effectively evaluate this variation, previous studies used the profilometer technique [7]. Although overlaid and non-overlaid composite panels (MDF-PB) that were manufactured from eastern redcedar were evaluated, there is insufficient information on surface roughness properties of overlaid and non-overlaid Eastern redcedar PB and MDF in the form of sandwich configuration. Therefore, the objective of this study was to evaluate the surface roughness and Janka hardness values of experimentally manufactured wood composite panels at 40%, 60% and 95% humidity levels. Data and conclusions drawn from this research would reveal a more efficient utilization of composite panel products for a variety of applications. 

## 2. Materials and Methods

### 2.1. Panel Manufacture

**Figure 1:**
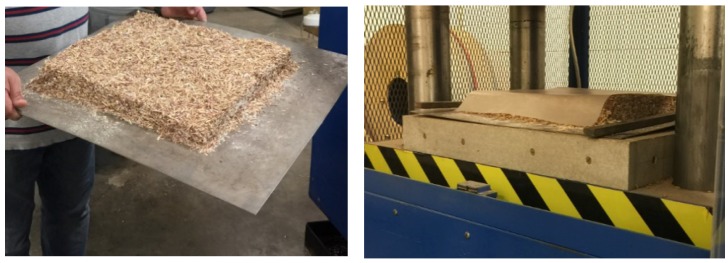
Manufacturing of Panel Samples.

Eastern redcedar (*Juniperus virginiana* L.) particles was supplied by a local sawmill in Oklahoma City. The particles contained both heartwood and softwood fractions of the trunk from eastern redcedar trees. Particles were dried to 2–3% moisture content in a laboratory type oven with a 1.0 m^3^ at the temperature of 67 ± 2 °C for 72 h. Dried particles were classified into two particle sizes, namely fine and coarse, on a 1 mm screen and 3 mm, respectively. After screening urea formaldehyde (UF) was blended with particles. Experimental panels were compressed at a temperature of 180 ± 2 °C and a pressure of 5.17 MPa for 5 min. All of the panels were pressed to a nominal thickness of 14 mm, and their target density was 0.70 g/cm^3^. Panels that were prepared with a length of 50 cm, width of 50 cm, and thickness of 14 mm. Manufacting process of composite panels is illustrated in Figure 1. 

### 2.2. Roughness Test of Samples

Stylus method is a well accepted technique, resulting in quantitavie numerical values on the surface of sample. The profilometer consists of main unit and pick up which has a skid-type diamond stylus with 5 m tip radius and tracing span constant speed of 1 mm/s over 15.2 mm at a surface. Technical details and working principles of stylus type profilometer are presented in a past work [18]. Roughness parameters, such as average roughness (R*a*), mean peak-to-valley height (R*z*), and maximum roughness (R*max*), can be calculated from the digital information [18]. 

**Figure 2:**
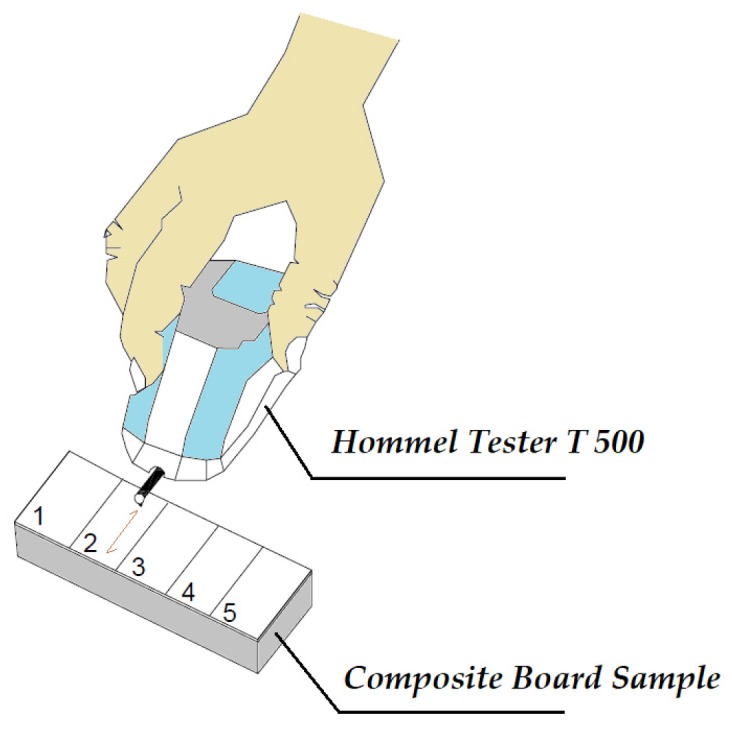
Roughness Test of Composite Panels Using Stylus method.

The calibration of the profilometer was checked every 100 measurements by using a standard reference plate with R*a* values of 3.02 μm. Samples with the size of 12 cm by 5 cm were used for random roughness measurements for test. A total of 20 samples were used for roughness measurements as illustrated in Figure 2. 

### 2.3. Hardness Test of Samples

**Figure 3:**
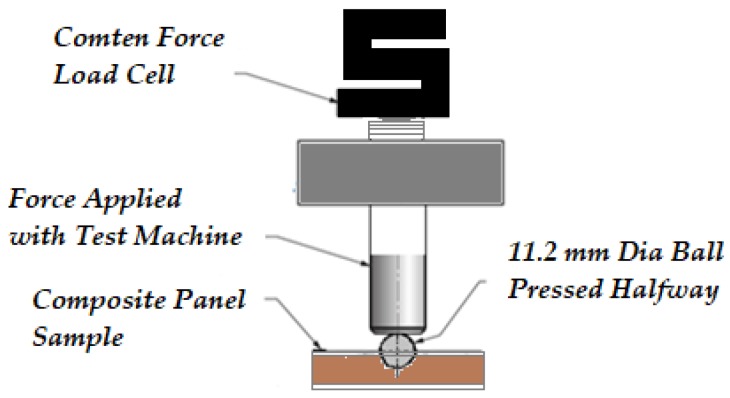
Janka hardness measurement with Comten 95 Series Universal Testing machine.

Hardness of the non-overlaid, overlaid particleboard, and non-overlaid, overlaid fiber sandwich panel specimens was tested by embedding a hemisphere steel having 11.2 mm diameter onto their tangential surface using a Comten 95 Series Universal Testing machine. Five measurements were taken from each sample and recorded in kg to evaluate their Janka hardness, as illustrated in Figure 3 [18].

### 2.4. Overlaying of Samples and Relative Humidity Exposure

**Figure 4:**
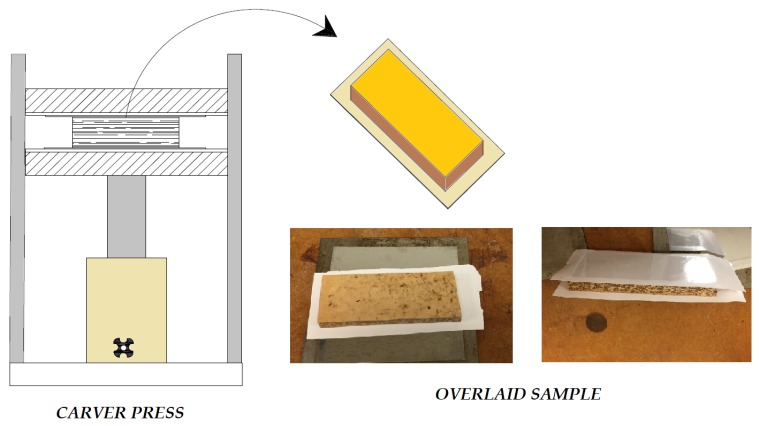
Overlaying Process.

A total of ten, five for each type of composites with dimensions of 12 cm by 5 cm were overlaid with melamine based decorative paper having weight of 7000 g/cm^2^. The test samples overlaid 50 s at Carver press with a 165 ± 5 °C under pressure of 2.3 MPa. Test samples were conditioned in a chamber with a temperature of 20 °C and a relative humidity of 65% until they reach to the equilibrium moisture content before any roughness measurements were taken from their surface. The overlaying process of the specimens is shown in Figure 4. 

**Figure 5:**
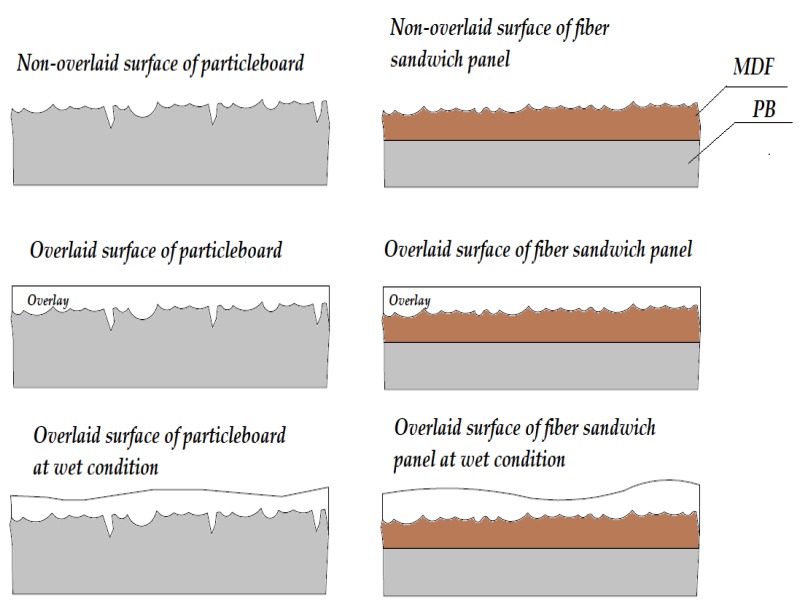
Penetration of humidity on surface characteristics.

After initial measurements were taken from surface of samples, they were placed in chamber having 95% relative humidity and were kept for 10 days. In the next step individual samples were weighted at an accuracy of 0.1 g. Later roughness measurements from the surface of each overlaid samples. Roughness values of the samples were quantitatively evaluated at initial dry condition and as they were exposed to relative humidity levels of 60% and 95%. Penetration of humidity on roughness of overlaid and non-overlaid substrate is schematically illustrated in Figure 5.

### 2.5. Analysis of Data

In order to analyze the significant differences of all the parameters that were used in this study, one-way analysis of variance (one-way ANOVA) was used. All results were computed employing IBM Statistical Package for the Social Sciences software (SPSS), version 21 (IBM Corporation, North Castle, NY, USA).

## 3. Results and Discussion

Measurements of average surface roughness values of fiber-sandwich composite panels are illustrated in Table 1 and Table 2, measurements of average surface roughness of particleboard panels are displayed. Overlaid samples having fiber layers on their surface had an average R*a* value of 0.48 μm once they were exposed to 60% and 95% relative humidity levels, corresponding values were 0.62 μm and 1.67 μm, respectively. 

As expected, relative humidity effects all three measures of surface roughness in both fiber sandwich boards. It is seen in Table 1 a trend in roughness values appearing. As relative humidity increases from 40% to 95%, non-overlaid fiber sandwich boards average surface roughness increases two-fold. This trend holds similarly for R*z* and R*max* as well.

It seems that PB samples had rougher initial average surface values, however they had similar trend having higher R*a* values once they were exposed to 60% and 95% relative humidity levels, as illustrated in Table 2.

### 3.1. Effect of the Relative Humidity Level on Roughness Values of Fiber-Sandwich Panels

In Table 3, ANOVA results related to the effectiveness of humidity level on surface roughness and Janka hardness values of the samples were discussed. 

Based on ANOVA, significant difference was observed between main effects such as composite panel types and humidity levels at a 95% confidence level. The average surface roughness values were found to be effective (*p* < 0.05) on interaction effects between panel types and humidity level. 

After relative humidity exposure process, the surface roughness of composite panel specimens increased from 3.22 μm to 5.82 μm and 80% increase in the surface roughness was found. Table 4 also displays the results from the Duncan test that are related to the homogeneous subsets according to the values determined in this work. Homogeneity group values-A of 0.92 μm and 3.22 μm were determined for material types, relative humidity levels, respectively.

### 3.2. Evaluation of Surface Roughness Values at Fiber-Sandwich Panels

**Figure 6:**
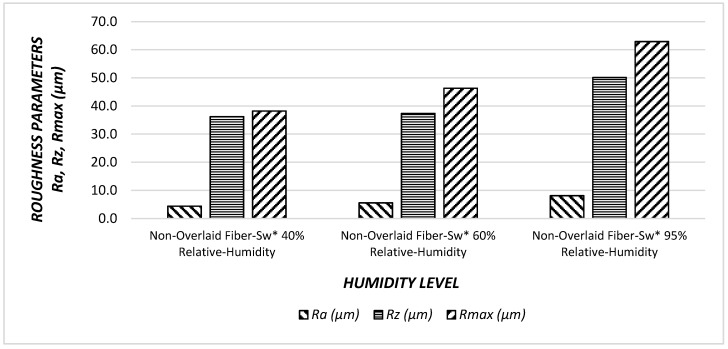
Surface roughness values (R*a*, R*z*, R*max*) of the non-overlaid fiber sandwich panels (Fiber-Sw*) under effect of humidity.

**Figure 7:**
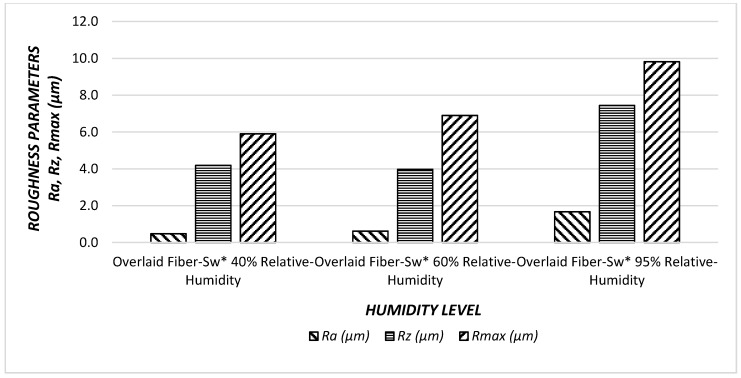
Surface roughness values (R*a*, R*z*, R*max*) of the overlaid fiber sandwich panels (O*-Fiber Sw*) under effect of humidity.

Both fiber-sandwich type and particleboard samples had higher surface roughness values at 95% humidity level. It appears that humidity level exposure effected surface quality of fiber-sandwich panels. In Figure 6 and Figure 7, the relationships between surface roughness parameters and humidity levels are illustrated. 

It can be noticed that as percentage of relative humidity increase, all of the surface roughness values increase. Specifically, R*a* had the greatest increase with a value of 83%, followed by R*max* 64%, and finally R*z* the least 38% increase. If the trends are compared between the surface roughness characteristics, *Ra* had a steeper slope than that of other parameters. Therefore, it appears that an increase in relative humidity adversely influenced overall surface quality of the samples. 

Increasing the percentage of relative humidity increased all the parameters of the surface roughness; this difference is due to the overlay, which protects the surfaces against relative humidity. Using overlay on the fiber sandwich board surfaces reduces the negative effects of relative humidity with an approximate value of nine times. 

### 3.3. Surface Roughness Values of Particleboard Panels

**Figure 8:**
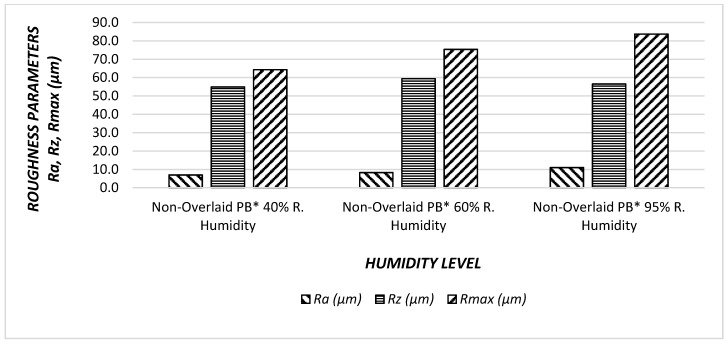
Surface roughness values (R*a*, R*z*, R*max*) of the non-overlaid particleboard (PB). Under effect of humidity.

**Figure 9:**
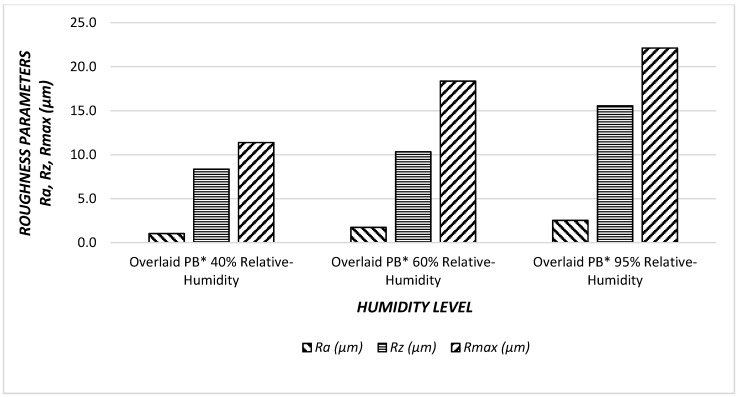
Surface roughness values (R*a*, R*z*, R*max*) of the overlaid particleboard (O*-PB) under effect of humidity.

Both MDF-Sandwich panel samples had a higher surface roughness values at 95% humidity level. Humidity levels effected MDF sandwich panels directly and surface quality deteriorated with humidity. In Figure 8 and Figure 9, the relationships between surface parameters and humidity levels are illustrated.

The percentage of relative humidity increase, all surface roughness values of non-overlaid particleboard increase. Specifically, R*a* had the greatest increase with a value of 58%, followed by R*max* 30%, and finally R*z,* with the least 8% increase. If the trends are compared between the surface roughness characteristics, *Ra* had a steeper slope than that of other parameters. Therefore, it seems that increase in relative humidity adversely influenced the overall surface quality of the samples.

All of the surface roughness values of overlaid particleboard increase after increasing relative humidity. Specifically, R*a* had the greatest increase with a value of 143%, followed by R*max* 96%, and finally, R*z* the least, with an 86% increase. If the trends are compared between the surface roughness characteristics, R*a* had a steeper slope than that of other parameters. Therefore, it appears that the increase in relative humidity adversely influenced all the measures of surface quality for each sample evaluated. Increasing the percentage of relative humidity increased all parameters of the surface roughness, this difference is due to the overlay, which protects the surfaces against relative humidity. Using overlay on the particleboard surfaces reduces the negative effects of relative humidity, with an approximate value of six times. 

### 3.4. Results of Janka Hardness Values

**Figure 10:**
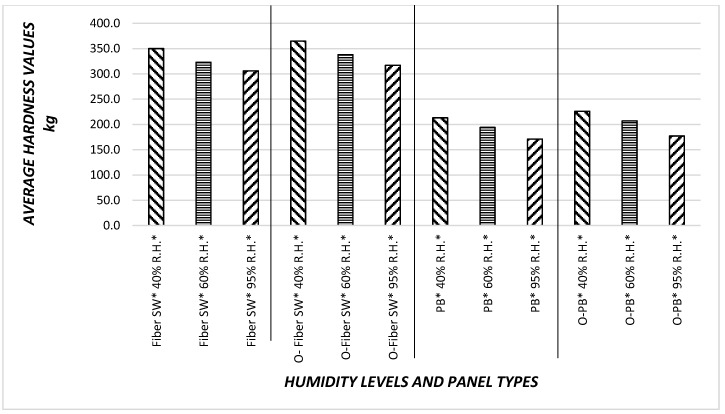
Average hardness values of composite panels.

Figure 10 illustrates overall hardness values of the samples. The highest Janka hardness value of 365- kg was found for overlaid fiber sandwich panel samples. Once these specimens were exposed to 60% and 95% relative humidity levels sequentially, their hardness characteristics were inversely influenced, as can be observed from Figure 10. In both overlaid samples were recorded slightly higher hardness values. This can be attributed to the brittleness of the overlay paper. Particleboard samples resulted in relatively lower hardness values as compared to those of sandwich type panels. The density of face layers of sandwich type samples having compact thin layers could be a reason for such findings. Lower hardness of particleboard samples could also be due to their single layer configuration. Similar to sandwich type panels, overlaid particleboard specimens also had slightly enhanced hardness values, which can also be related to the brittleness of overlay paper. 

As percentage of relative humidity increase, all Janka hardness values decrease. Specifically, non-overlaid particleboard had the minimum Janka harness value 171 kg overall. If the Janka hardness values are compared between the overlaid and non-overlaid specimens, overlaid samples have better Janka hardness values between 5% and 6%. Therefore, it appears that the increase in relative humidity adversely influenced the overall hardness values of the specimens. 

## 4. Conclusions

This study aimed to quantify the effect of humidity exposure on both surface quality and Janka hardness for different types of wood composite material, namely these were overlaid and non-overlaid composite panels. Based on the findings that are discussed in this work, as a conclusion, overlaid samples that were exposed to different levels of relative humidity had less overall surface roughness characteristics than the non-overlaid samples. Furthermore, the Janka hardness results were higher in the case of overlaid samples than in the non-overlaid samples. The data found in this work can inform future decisions to make more efficient use of overlaid and non-overlaid wooden composite boards. This work can be extended in further studies by evaluating other mechanical properties, such as bending and compression strength, of wood composite boards under different levels of relative humidity. Additionally, through future studies of dimensional stability, as effected by relative humidity, can gain a better understanding of the behavior of overlaid panels. 

## Figures and Tables

**Table 1:** Average surface roughness (R*a*) values of fiber-sandwich composite panels.

Panel Types	Statistical Value	Surface Roughness		
		R*a* (μm)	R*z* (μm)	R*max* (μm)
Non-Overlaid Fiber-*Sw* * 40% Relative-Humidity	Mean	4.41	36.20	38.19
	Standard Err.	0.77	10.83	6.59
Non-Overlaid Fiber-*Sw* * 60% Relative-Humidity	Mean	5.57	37.35	46.29
	Standard Err.	1.16	8.46	10.80
Non-Overlaid Fiber-*Sw* * 95% Relative-Humidity	Mean	8.10	50.12	62.88
	Standard Err.	1.92	9.46	12.49
Overlaid Fiber-*Sw* * 40% Relative-Humidity	Mean	0.48	4.19	5.90
	Standard Err.	0.13	2.49	4.05
Overlaid Fiber-*Sw* * 60% Relative-Humidity	Mean	0.62	3.97	6.90
	Standard Err.	0.19	2.45	5.04
Overlaid Fiber-*Sw* * 95% Relative-Humidity	Mean	1.67	7.44	9.82
	Standart Err.	0.71	3.40	4.08

* *Sw*: Sandwich Panel

**Table 2:** Average surface roughness (R*a*) values of particleboard panels.

Panel Types	Statistical Value	Surface Roughness		
		R*a* (μm)	R*z* (μm)	R*max* (μm)
Non-Overlaid PB * 40% Relative-Humidity	Mean	6.95	54.84	64.31
	Standard Err.	1.13	10.14	9.75
Non-Overlaid PB * 60% Relative-Humidity	Mean	8.24	59.37	75.34
	Standard Err.	1.20	8.50	9.83
Non-Overlaid PB * 95% Relative-Humidity	Mean	10.99	56.50	83.71
	Standard Err.	1.80	15.66	8.70
Overlaid PB * 40% Relative-Humidity	Mean	1.04	8.36	11.38
	Standard Err.	0.34	4.76	4.73
Overlaid PB * 60% Relative-Humidity	Mean	1.74	10.32	18.37
	Standard Err.	0.80	5.00	8.63
Overlaid PB * 95% Relative-Humidity	Mean	2.53	15.56	22.12
	Standart Err.	0.72	10.19	12.22

* PB: Particleboard

**Table 3:** ANOVA results related to the effect of average surface roughness *Ra* level. based on the humidity levels and wooden composite panels.

Applied Tests	Mean Square	F Value	Level of Significance (*p* ≤ 0.05)
Intercept	5717.19	5067.70	0.000
Panel Types	3027.47	894.51	0.000
Relative Humidity Levels	353.75	156.78	0.000
Panel Types x Relative Humidity Level	85.88	12.68	0.000

**Table 4:** Comparative test results for the effect of relative humidity level on various properties of the composite panel samples for homogeneity groups.

Parameters	Groups	HG. * A	HG. * B	HG. * C	HG. * D
Materials	Overlaid Fiber-*Sw* *	0.92	-	-	-
	Overlaid Particleboard	-	1.77	-	-
	Non-Overlaid Fiber-*Sw* *	-	-	6.02	-
	Non-Overlaid PB *	-	-	-	8.73
Relative Humidity Levels	40% Relative Humidity	3.22	-	-	-
	60% Relative Humidity	-	4.04	-	-
	95% Relative Humidity	-	-	5.82	-

* HG: Homogeneity groups, * *Sw*: Sandwich panel, * PB: Particleboard, all result are R*a* values.
